# Fingolimod induces neurogenesis in adult mouse hippocampus and improves contextual fear memory

**DOI:** 10.1038/tp.2015.179

**Published:** 2015-11-24

**Authors:** P Efstathopoulos, A Kourgiantaki, K Karali, K Sidiropoulou, A N Margioris, A Gravanis, I Charalampopoulos

**Affiliations:** 1 Department of Pharmacology, School of Medicine, University of Crete, Heraklion, Greece; 2 Institute of Molecular Biology & Biotechnology, Foundation of Research & Technology-Hellas (IMBB-FORTH), Heraklion, Greece; 3 Department of Biology, University of Crete, Heraklion, Greece; 4 Department of Clinical Chemistry, School of Medicine, University of Crete, Heraklion, Greece

## Abstract

Fingolimod (FTY720) was the first per os administered disease-modifying agent approved for the treatment of relapsing–remitting multiple sclerosis. It is thought that fingolimod modulates the immune response by activating sphingosine-1 phosphate receptor type 1 (S1P1) on lymphocytes following its *in vivo* phosphorylation. In addition to its immune-related effects, there is evidence that fingolimod exerts several other effects in the central nervous system, including regulation of the proliferation, survival and differentiation of various cell types and their precursors. In the present study, we have investigated the effect of fingolimod on the production of new neurons in the adult mouse hippocampus and the association of this effect with the ability for pattern separation, an established adult neurogenesis-dependent memory function. Immunofluorescence analysis after chronic administration of a physiologic dose of fingolimod (0.3 mg kg^−1^) revealed a significant increase in both the proliferation and the survival of neural progenitors in the area of dentate gyrus of hippocampus, compared with control animals. These effects were replicated *in vitro*, in cultures of murine hippocampal neural stem/precursor cells that express S1P1 receptor, suggesting cell-autonomous actions. The effects of fingolimod on neurogenesis were correlated to enhanced ability for context discrimination after fear conditioning. Since impairment of adult hippocampal neurogenesis and memory is a common feature of many neuropsychiatric conditions, fingolimod treatment may be beneficial in therapeutic armamentarium of these disorders.

## Introduction

Recent research evidence indicates that neurogenesis is an important player in the plasticity of adult mammalian brain, responsible for several physiological functions including learning and memory.^1, 2^ The production rate of new neurons during adulthood, from endogenous neural stem cells (NSCs), takes place primarily in the subgranular zone (SGZ) of the hippocampal dentate gyrus (DG) and in the subventricular zone.^3^ The processes of proliferation, migration and differentiation of NSCs into functionally mature neurons, which get integrated to the existing neural circuit, is regulated by a plethora of intrinsic, as well as extrinsic stimuli.^3^ Neurogenesis declines with advancing age in parallel to the decline of several mental faculties including cognition and memory.^4, 5^ In addition, decline of adult hippocampal neurogenesis is a common feature of several neurodegenerative diseases both in humans and in rodents, possibly as an homeostatic attempt of central nervous system (CNS) to preserve its own cellular and functional integrity.^6, 7^ Thus, various strategies have been employed to enhance this endogenous mechanism of self-repair and alleviate linked cognitive symptoms.

Fingolimod (FTY720; Gilenya, Novartis Pharma, Basel, Switzerland) is a sphingosine-1-phosphate (S1P) analog, approved as an oral therapeutic agent for relapsing–remitting multiple sclerosis (MS). MS is a chronic autoimmune demyelinating disorder, which leads to neurodegeneration and brain atrophy in various regions including the hippocampus^7^ and is accompanied by physical disability and cognitive decline, with memory loss being one of its major manifestations. Fingolimod exerts its beneficial effects on relapsing–remitting MS by sequestering lymphocytes within the lymph node.^8^ However, there is increasing evidence suggesting that fingolimod also affects the function of various cell types in the CNS including astrocytes, oligodendrocytes, neurons and their progenitors.^9, 10, 11^

Fingolimod, after its *in vivo* phosphorylation to fingolimod-phosphate (fingolimod-p), mimics the actions of the endogenous S1P, which belongs to lysophospholipids, a class of membrane-derived bioactive lipid mediators, which acts by activation of five types of GPCR trans-membrane S1P receptors (1–5), but also as an intracellular second messenger itself. Among these receptors, the S1P type 1 receptor (S1P1) appears to represent the most prominent mediator of fingolimod effects both in the CNS and the periphery.^12, 13, 14^ In mouse, the S1P1 was found to be mainly located adjacent to lateral ventricles during development,^15^ while S1P was shown to increase GTPγS binding via activation of G proteins in the subventricular zone,^16^ suggesting that it might exert a role in the control of neurogenesis. The neurogenic effects of endogenous S1P were also shown in sphingosine kinase-null mice, which are characterized by severely disturbed neurogenesis, increased apoptosis and decreased mitosis in the developing CNS, resulting in embryonic lethality.^15^ Moreover, S1P1 receptor-null mice show severe defects in neurogenesis, suggesting that the mechanism by which S1P promotes neurogenesis is, partially at least, mediated by the S1P1 subtype.^17^ Finally, S1P induces the proliferation and morphological changes of embryonic hippocampal neural progenitors in cultures, through ERK signaling.^16^

Despite the extensive literature discussing the involvement of S1P signaling in brain development, little is known about its role in adult neurogenesis. Furthermore, the potential functional implications of the actions of S1P receptor modulator, fingolimod, on adult neural progenitors, have been neglected so far. Thus, in the present study we sought to test the effects of fingolimod on adult murine hippocampal neurogenesis and its potential involvement in memory function.

## Materials and methods

### Animals

Male C57/BL6 mice were maintained on a 12-h light/dark cycle (lights on at 0700 hours) with *ad libitum* access to food and water. Animals were habituated to housing conditions for 1 week prior to the beginning of the experimental procedures. Animal experimentation received the approval of Veterinary Directorate of Prefecture of Heraklion, Crete and was carried out in compliance with Greek Government guidelines and the guidelines of FORTH ethics committee.

### Tissue processing and immunofluoresence

Mice were killed with pentobarbital and trans-cardially perfused with saline followed by 4% paraformaldehyde (Sigma-Aldrich, St. Louis, MO, USA) in phosphate buffered saline (PBS). The brains were dissected and post-fixed overnight in the same solution at 4 ^o^C. After thorough washing in PB 0.1 M, brains were cryoprotected by being immersed in 30% sucrose solution in PB 0.1 M for 24 h at 4 ^o^C until they sunk and were frozen in isopentane at−40 ^o^C. Coronal sections of 40 μM were cut in the dorsoventral axis of hippocampus (from bregma −1.34 mm to −3.80) and stored in cryoprotective medium (30% glycerol/30% ethylene glycol in 50 mM phosphate buffer) at−20 °C until they processed for immunofluoresence.

### NS/PCs cultures

The hippocampi of postnatal day 7 (P7) C57/BL6 mice were digested for 30 min in accutase solution (Sigma-Aldrich) at 37 ^o^C. After mechanical dissociation, cells were plated at a density of 5 × 10^4 ^ml^−1^ into uncoated T25 culture flasks in Dulbecco’s Modified Eagle’s Medium/Nutrient Mixture F-12 Ham (Sigma-Aldrich) supplemented with 1% B27 (Invitrogen, Thermo Fisher Scientific, Waltham, MA, USA), L-glutamine 2 mM (Gibco, Thermo Fisher Scientific), D-glucose 0.6%, primocin 100 μg ml^−1^ (Invitrogen), in the presence of 20 ng ml^−1^ FGF2 (R&D Systems, Minneapolis, MN, USA) and EGF (R&D) and allowed to form neurospheres. Cells were passaged every 5th day by dissociating neurospheres into single cells with accutase (Sigma-Aldrich).

### NS/PCs proliferation and survival assay

For the proliferation assay, passages 1–3 of primary neurospheres were dispersed to single cell suspension and were plated in Poly-D-Lysine/Laminin-coated dishes and left to stay for 24 h in complete medium. The day after cells were deprived of EGF/FGF for 3 h and then exposed for 24 h to 1 μM fingolimod-p in the presence or absence of 300 ng ml^−1^ pertussis toxin (PTX) or 10 μM of ERK1/2 inhibitor U0126 that were added 30 min or 3 h before drug addition. At the end of the treatments, 10 mM BrdU (5-bromo-2′-deoxyuridine) was applied to the cultures for 16 h to label all actively proliferating cells before they were fixed and stained against BrdU. For the assessment of the effects of fingolimod on survival of NPCs after growth factors withdrawal, cells were plated as above and cultured for 72 h in EGF/FGF-free medium in the presence or absence of 1 μM fingolimod-p, with or without the addition of 10 μg ml^−1^ anti-BDNF-neutralizing antibody. Finally, cells were fixed and stained with *in situ* cell death detection labeling kit (terminal deoxynucleotidyl transferase dUTP nick end labeling (TUNEL); Roche, Basel, Switzerland) according to the manufacturer's instructions.

### Immunofluoresence

Free-floating sections or neural stem/precursor cells (NS/PCs) attached in Poly-D-Lysine/Laminin and fixed with 4% paraformaldehyde solution for 15 min were washed twice with 0.1 M PB pH 7.4 and blocked in 5% BSA in 0.3% Triton X-100 in PBS (PBST) for 1 h at room temperature before they were incubated with primary antibodies in 0.3% PBST overnight at 4 ^o^C. The day after sections or cells were washed twice with PBS and incubated with Alexa Fluor chromophore-conjugated secondary antibody (1:1000 dilution, Invitrogen) in 0.3% PBST for 1 h at room temperature. Finally, sections were washed twice again with PBS and mounted onto slides using antifade reagent with DAPI (4′,6-diamidino-2-phenylindole, Dilactate; Invitrogen). For detection of BrdU-labeled nuclei, specimens have been previously incubated in 2 N HCl at 37 ^o^C, followed by a 10-min rinse in 0.1 M sodium tetraborate pH 8.5 and two rinses with PBS before blocking step.

### Cell counts and quantification

Based on a modified unbiased stereology protocol,^18, 19^ one out of every six adjacent sections was chosen and processed for BrdU immunohistochemistry. The number of BrdU+ cells was then counted under × 40 magnification under a fluorescent microscope (Leica DMLB equipped with a DC300 F camera (Leica Microsystems CMS, Mannheim, Germany) at the area of granular cell layer and SGZ (defined as a two cell layer in the borders of granular cell layer, omitting these in the outermost focal plane) for a total of 10 sections and the average number of cells was estimated. The mean was then multiplied with the total number of sections (~60) to estimate the total number of cells per DG.

To measure the total volume of DG, the area of granular cell layer was outlined and computed using images in every 6th adjacent section for a total of 10 sections in photos taken by a confocal fluorescence microscope (Leica TCS SP2 (Leica Microsystems CMS)). Subsequently, and according to the Cavalieri principle, the areas were summed and multiplied with the intersection distance and average section thickness to estimate the entire DG volume in mm.^3^

For *in vivo* co-localization experiments, confocal analysis under × 63 oil lenses was conducted. The percentage of double-labeled cells for the each marker was estimated by counting the cells in the DG in three sections of total five mice.

For *in vitro* experiments, the number of BrdU- or TUNEL-positive cells in each culture was counted using an objective (× 32) from 8–10 visual fields for every condition. DAPI-stained cells represent the total cell number. Finally, the percentage of BrdU+/DAPI+ or BrdU+/TUNEL+ cells was normalized to the control condition. Mean was estimated for each condition from three independent experiments.

### Western blot analysis and ELISA

Cells were harvested in lysis buffer (50 mM Tris-HCl, 0.15 M NaCl, 1% Triton X-100, pH 7.4) and supplemented with protease inhibitors (1 mM PMSF and 1 g ml^−1^ aprotinin). Total protein was quantified with BCA protein kit (Pierce, Thermo Fisher Scientific, Rockford, IL, USA) and 30 μg of total protein was loaded and run on sodium dodecyl sulfate–PAGE. The proteins were then transferred to a nitrocellulose membrane and blocked in a solution of 5% BSA and 0.1% Tween-20 in TBS (TBS-T) for 1 h followed by incubation with the primary antibody in blocking solution overnight at 4 ^o^C. The next day, the membranes were washed three times in TBS-T and incubated in anti-rabbit secondary antibody conjugated to horseradish peroxidase (1:5000) in blocking solution for 1 h. Finally, the membranes were washed again in TBS-T and incubated for detection with enhanced chemiluminescence substrate (Pierce).

For BDNF expression in hippocampal NS/PCs, each protein-sample was quantified with specific mouse BDNF enzyme-linked immunosorbent assay (ELISA) kit (Boster Immunoleader, Pleasanton, CA, USA) according to manufacturer's instructions and normalized to pg per mg of total protein. Then, relative BDNF expression was normalized to the control conditions in each experiment. Mean values were estimated from three different cultures.

### Antibodies and reagents

The following primary antibodies and dilutions were used: rat anti-BrdU (1:300, Abcam, Cambridge, UK); rabbit monoclonal anti-EDG1 (1:1000, Abcam); goat anti-DcX,(1:150, Santa Cruz Biotechnology, Dallas, TX, USA); mouse anti-NeuN (1:200, Chemicon, Billerica, MA, USA); rabbit polyclonal anti-p-p44/42 and p44/42 MAPK (1:1000, Cell Signaling, Danvers, MA, USA).

The following reagents were used in proliferation and survival assay: fingolimod, fingolimod-phosphate (Novartis, Basel, Switzerland), Human BDNF (Novus Biologicals, Iowa City, IA, USA), Anti-BDNF (mab#9 Developmental Studies Hybridoma Bank, Iowa City, IA, USA), U0126 (Sigma-Aldrich) and PTX (Sigma-Aldrich).

### Contextual fear discrimination

Male mice, 3-months-old, received either a vehicle or fingolimod treatment for 14 days, once a day, 0.3 mg kg^−1^ per day, i.p. On the 15th day, mice (one at a time) were placed in the fear conditioning chamber (MedAssociates, St. Albans, VT, USA), which was controlled through a custom-made interface connected to the computer (both saline-treated and drug-treated animals were present in each cage and at every single day of behavioral experimentation, both groups of animals were used). After 7 min of habituation to the conditioning chamber, each mouse received one mild electrical foot shock (600 ms, 0.75 mA), and remained in the chamber for another 5 min.^20^ The following day, mice were returned to the training chamber using the same context (context A) for 3 min. Subsequently, they were removed from the chamber, and after 1 h of rest they were placed in the same chamber in which the context was modified by the addition of olfactory auditory and visual cues (mild scent on the walls, open fan and red floor) for another 3 min. The same procedure was repeated the next day. Mice in the no-shock group followed the same protocol as above, but did not receive any electrical foot shock during the training day. The freezing behavior was analyzed manually using the J-Watcher software (http://www.jwatcher.ucla.edu/). Context discrimination ratio is defined as (Freezing in context A−Freezing in context B)/(Freezing in context A+Freezing in context B).

### Statistical analysis

Statistical analysis was performed using Graphpad Prism software (La Jolla, CA, USA*)* or Microsoft Excel. Statistical significance was assessed by unpaired two-tailed Student’s *t*-tests, analysis of variance (ANOVA) followed by Fisher’s predicted least-square difference *post hoc* tests when necessary or two-way-ANOVA with fingolimod treatment and age as the factors followed by Bonferroni *post hoc* tests when appropriate. Sample size required in each case was estimated based on the number of groups and the expected effect size with G*power statistics software (Düsseldorf, Germany). Statistical significance was established at *P*⩽0.05.

## Results

### Fingolimod-induced adult neurogenesis in young but not in aged mice

**Figure 1:**
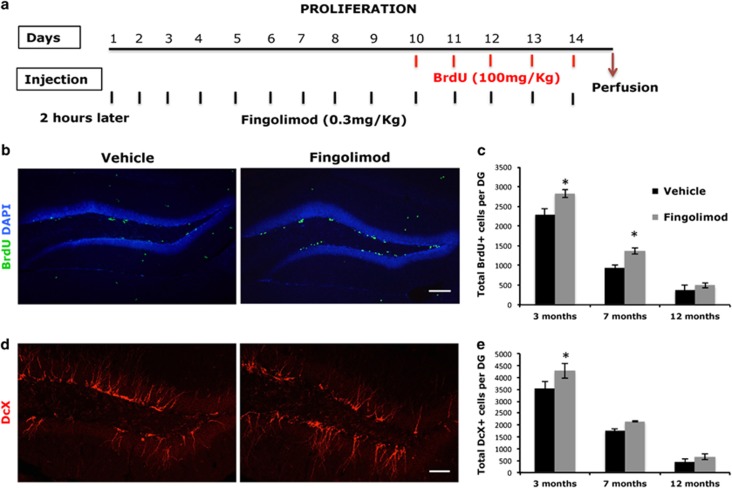
(**a**) Illustrative graph that shows the exact time of administration of BrdU and fingolimod in animals for the NSCs proliferation experiments. (**b**) Coronal section of dorsal DG from 3-month-old C57/BL6 mice injected with fingolimod or vehicle daily for 14 days, co-injected with BrdU for the last 5 days. Sections were immunostained for BrdU (green) and counterstained with DAPI. (**c**) Quantification of BrdU+ cells in SGZ in Fingolimod or vehicle-treated mice of different ages. (**d**) Coronal section of dorsal DG from 3-month-old C57/BL6 mice injected with Fingolimod or vehicle daily for 14 days. Image depicts DcX (red) immunostained immature neurons. (**e**) Quantification of DcX cells in C57/bl6 mice of different ages treated with fingolimod or vehicle for 14 days. Scale bar, 150 μm (mean±s.d.; *n*=6; *P<0.05 vs vehicle). BrdU, 5-bromo-2′-deoxyuridine; DAPI, 4′,6-diamidino-2-phenylindole; DcX, doublecortin; DG, dentate gyrus; NSC, neural stem cells; SGZ, subgranular zone.

To test the effect of the chronic exposure to fingolimod on the proliferation of the NSCs in the adult and the aged DG, mice of three different age groups (3, 7 and 12 months) were administered i.p. 0.3 mg kg^−1^ per day of fingolimod or the vehicle for 14 days, while they were also administered 100 mg kg^−1^ of the thymidine analog BrdU during the last 5 days of treatment—to mark all proliferative cells at the S phase of cell cycle—and killed 24 h later (Figure 1a). Two-way ANOVA analysis for fingolimod treatment and age revealed that the number of proliferating, BrdU+, NS/PCs in the SGZ was significantly decreased with age (*P<*0.0001, F(2–34)= 1451.47), as it was expected. Fingolimod treatment, had a positive overall effect on the number of proliferating NS/PCs (*P<*0.0001, F(1–34)=120.98). Moreover, it was shown that there was a significant interaction between the fingolimod treatment and age (*P<*0.0001, F(2–34)=14.69), indicating that fingolimod differentially affected the young and aged animals. *Post hoc* analysis showed a significant increase in the number of proliferating, BrdU+, NS/PCs in the SGZ in 3- as well as 7-month-old mice, compared with the untreated control animals (2831±138 vs 2295±103 BrdU+ for 3-month-old and 1368±86 vs 939±78 BrdU+ for 7-month-old mice, *n*=7, mean±s.d., *P<*0.05) (Figures 1b and c). Furthermore, the total volume of DG is not affected by the treatment (0.615 mm^3^±0.012 vs 0.59±0.04 mm^3^, *P>*0.5), confirming an increase in the actual number of proliferative cells. However, fingolimod did not have any significant effect on the proliferation of NSCs in 12-month-old animals (495±64 BrdU+ vs 378± 125 BrdU+ cells *n*=7, mean±s.d*. P<*0.05) compared with the controls of the same age (Figure 1c).

To assess the effects of the drug in net neurogenesis, the number of the new neurons was stained against the microtubule protein doublecortin (DcX) in the area and was counted in the same groups of mice. Stereological analysis showed that fingolimod significantly increased the amount of DcX+ cells only in young 3-month-old mice (4280±613 vs 3540±594, *P*<0.05), since *post hoc* analysis did not detect any significant effect at 7 or 12 months of age (2150±60 vs 2150±25 DcX+ and 670±126 vs 443±116, *P*>0.05). (Figures 1d and e).

**Figure 2:**
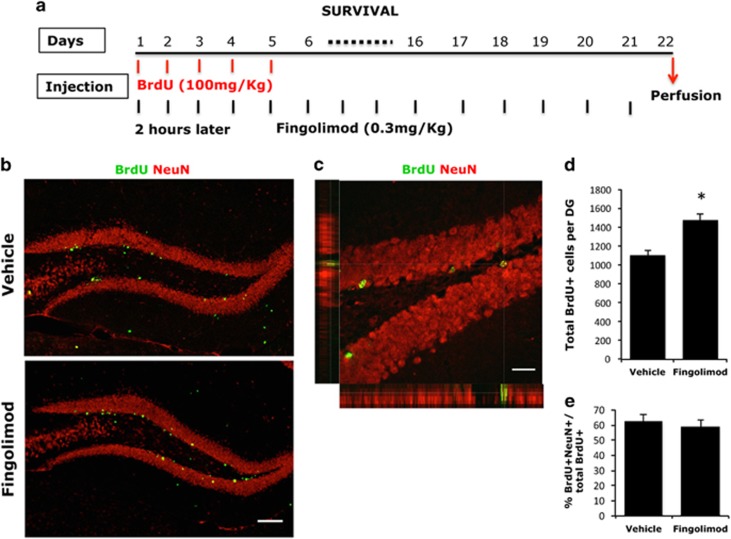
(**a**) Illustrative graph that shows the exact time of administration of BrdU and fingolimod in animals for the NSCs survival experiments. (**b**) Coronal section of dorsal DG from 3-month-old C57/BL6 mice injected with fingolimod or vehicle daily for 21 days, co-injected with BrdU for the first 5 days. Sections were immunostained for BrdU (green) and NeuN (red). (**c**) Higher magnification of a confocal image of BrdU- and NeuN-stained cells in SGZ. (**d**) Quantification of BrdU+ cells in SGZ and GCL in fingolimod or vehicle-treated 3-month-old mice (mean±s.d.; *n=*6; *P<0.05 vs vehicle)*.* (**e**) Percentage of BrdU+/NeuN+ cells in the DG of fingolimod or vehicle-treated mice 21 days after first BrdU administration. BrdU, 5-bromo-2′-deoxyuridine; DG, dentate gyrus; NSC, neural stem cells; SGZ, subgranular zone.

In a subsequent step, we examined the efficacy of fingolimod on the survival rate of NS/PCs of the SGZ and the production of new neurons at the age group that responded to the drug, in parallel to its effect on NSCs proliferation. To this aim, we pulsed 3-month-old mice with BrdU for the first 5 days of a 3-week period, when mice were i.p. administered with 0.3 mg kg^−1^ of fingolimod (Figure 2). The number of BrdU+ NS/PCs that survived after 21 days of administration was increased by ~35% (1475±65, *n*=7 vs 1102±53; *n*=6, mean±s.d., *P<*0.05) (Figures 2b and d). Moreover, the majority of BrdU+ cells were also stained positive for NeuN, suggesting a neuronal phenotype (Figures 2c and e). The fact that BrdU+/NeuN+ cell percentage does not significantly differ between treated and untreated mice indicates that the increase in the number of BrdU+ cells after 21 days of treatment, reflects an increase in the production of new neurons induced by fingolimod.

### Fingolimod-induced proliferation of NS/PCs in culture through activation of MAPK pathway

**Figure 3:**
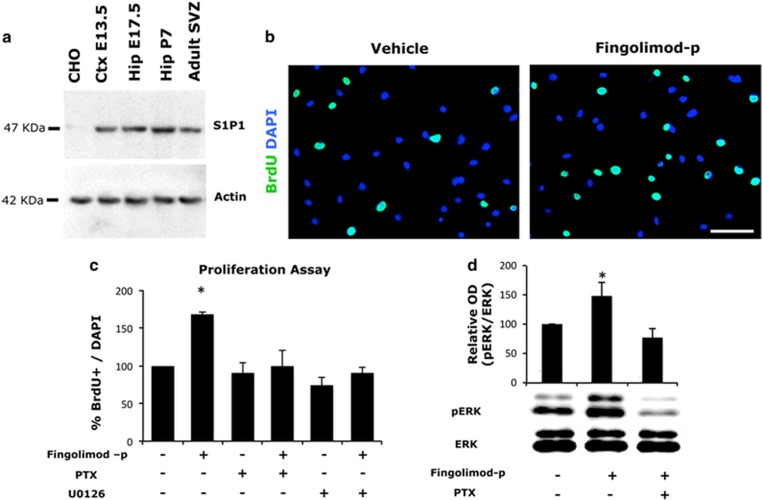
(**a**) Western blot analysis of S1P1 expression in neurospheres derived from the respective area as indicated and at specific ages (Ctx, cortical; Hip, Hippocampal; E, embryonic; P, postnatal). (**b**) BrdU staining of hippocampal neural stem cells after they have been treated for 24 h as indicated in the absence of growth factors. Scale bar, 100 μM and (**c**) percentage of BrdU+/DAPI+ (mean±s.d.; *n=*3; *P<0.05 vs vehicle-treated cells). (**d**) Phosphorylation of ERK1/2 after 10-min exposure of NS/PCs culture in growth factor free medium in the presence or absence of 1 μM fingolimod-p after they have been pre-incubated or not with PTX. Western blot analysis was performed and relative optical density (OD) of phosphorylated ERK1/2 (pERK) to this of total ERK1/2 (tERK) was estimated and normalized to control values (mean±s.d.; *n*=3; *P<0.05 vs vehicle-treated cells). BrdU, 5-bromo-2′-deoxyuridine; DAPI, 4′,6-diamidino-2-phenylindole; PTX, pertussis toxin.

To further explore the mitogenic effects of fingolimod that we observed *in vivo,* we used isolated NS/PCs derived from the hippocampus of C57/Bl6 mice of P7, which were grown as neurospheres in the presence of EGF/FGF. At this age, NSCs of the germinal zones acquire characteristics of adult NSCs while, at the same time, they yield the maximum number of progeny because of their high proliferative activity.^21^ These cells were found to specifically express the S1P1 receptor, as shown by western blot analysis of whole cellular extracts of neurospheres (Figure 3a). After dissociation, cells were plated as previously described in Materials and methods section, deprived of EGF for 3 h and exposed for 24 h to 1 μM fingolimod-p, which is the active metabolite of fingolimod *in vivo*. Finally, 10 mM BrdU was applied to the cultures for 16 h to label all actively proliferating cells. Treatment with fingolimod-p significantly increased the percentage of BrdU+ NS/PCs (Figures 3b and c). Moreover, since MAPK pathway induction—initiated from activation of cell surface receptor—has been widely implicated in the actions of both endogenous S1P, as well as fingolimod’s on various cell functions,^9, 11, 14^ we would like to test *in vitro* its involvement on the effects that we observed *in vivo*. Pre-incubation of culture media with either PTX (a well-known uncoupler of Gi protein from the enzyme adenylate cyclase) or a specific inhibitor of MEK1 (U0126) completely abolished the proliferative effects of fingolimod-p in postnatal hippocampal NS/PCs (Figure 3c). Furthermore, fingolimod-p was proved efficient to induce stimulation of ERK1/2 kinase in a PTX-sensitive manner (Figure 3d). The data above indicate that activation of S1P1 and subsequent stimulation of MAPK pathway is responsible for the mitogenic effects of fingolimod-p on postnatal NSCs.

### Fingolimod affected the survival of NS/PCs cells via an induction of BDNF expression

**Figure 4:**
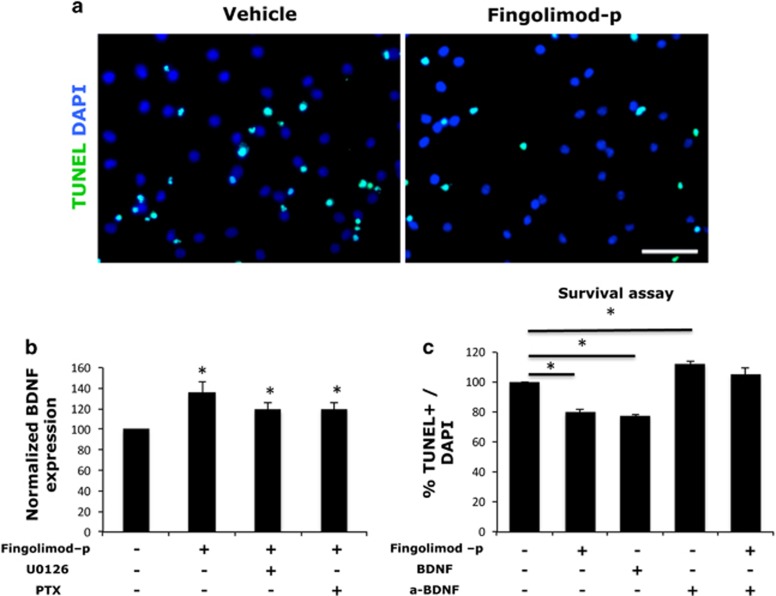
(**a**) TUNEL staining of hippocampal neural stem cells after 72 h treated as indicated in the absence of growth factors. (**b**) BDNF protein levels quantified by ELISA in lysates of hippocampal NSC cultures treated with 1 μM fingolimod-p or not in the presence or absence of U0126 or pertussis toxin for 24 h (mean±s.d.; *n*=3; *P<0.05 vs vehicle-treated cells). (**c**) Percentage of TUNEL+/DAPI+ (mean±s.d.; *n=*3; *P<0.05, vs vehicle-treated cells). Scale bar, 50 μM. DAPI, 4′,6-diamidino-2-phenylindole; ELISA, enzyme-linked immunosorbent assay; TUNEL, terminal deoxynucleotidyl transferase dUTP nick end labeling.

Recent findings support the idea that treatment with fingolimod increases the expression of BDNF in neurons *in vitro* and *in vivo*, thus promoting their survival.^22^ It is also known that NSCs are capable of secreting BDNF.^23, 24^ After a 24-h treatment with 1 μM fingolimod-p, we observed an increased production of BDNF from postnatal hippocampal NSCs in culture, which was not blocked by the use of U0126 or PTX (Figure 4b), indicating an S1P1-independent effect. In addition to this, fingolimod-p was able to decrease apoptosis rate in NS/PCs in culture after growth factor withdrawal for 72 h (Figure 4c), as it was revealed by TUNEL staining (Figure 4a). The pro-survival effects of fingolimod were attenuated by the use of an anti-BDNF neutralizing antibody showing that increased BDNF expression after fingolimod treatment is sufficient to protect these cells from apoptosis after growth factor withdrawal.

### Fingolimod treatment improved context discrimination

**Figure 5:**
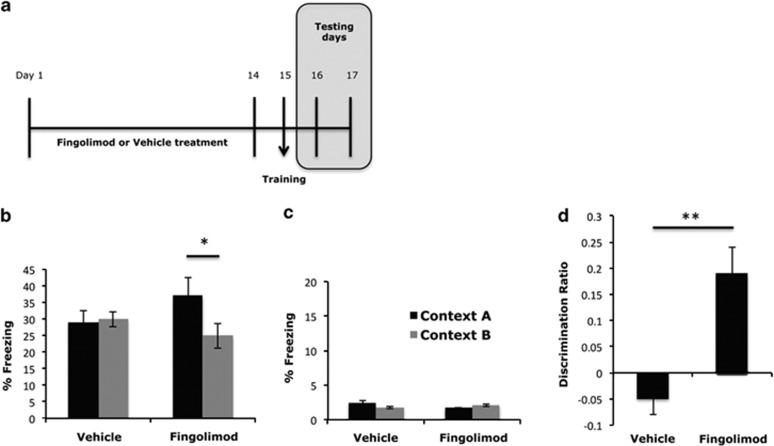
(**a**) Illustrative graph that shows the exact time periods for drug or vehicle administration, the training day and the testing days of the behavioral tasks. (**b**) Percent of time freezing in context A (black bars) and context B (gray bars) 1 day after fear conditioning, for mice that were treated with either vehicle or fingolimod for 14 days. (mean±s.e.m.; *n*=13; *P<0.05 vs freezing in context, A) (**c**) Percentage of freezing in context A and B for mice that have not received shock during the training day. (**d**) Discrimination ratio, which represent (freezing in context A−freezing in context B) to (freezing in context A+freezing in context B) (mean±s.e.m.; *n*=13; **P<0.01 vs vehicle).

Following these data, specific behavioral aspects of these fingolimod-induced changes were examined including changes of memory. To test the latter, we have used a context discrimination paradigm, which has been previously shown to reflect the functional role of hippocampal neurogenesis in the adults.^25^ According to this, adult (3-months-old) male mice were treated with fingolimod (0.3 mg kg^−1^ per day, i.p.) or vehicle for 14 days. On day 15 (the training day), mice were placed in the training chamber and received a mild foot shock (Figure 5a). On days 16 and 17, mice were returned either to the same context (context A) or to a slightly modified one (context B) and allowed to explore each context for 3 min. Subsequently, they were evaluated for their freezing behavior (Figure 5a). Mice that have been treated with fingolimod displayed a similar degree of freezing in context A, but significantly decreased freezing in context B (Figure 5b) (32.7%±3.5 vs 22.6%±3.7% freezing, *N*=13, mean±s.e.m., *P<*0.05). As a result, mice treated with fingolimod displayed significantly increased discrimination ratio (Figure 5d) (−0.05±0.03 vs 0.17±0.05, *N*=13, mean±s.e.m., *P<*0.05), which means that they could discriminate the similar context B better than mice that were treated with vehicle. Fingolimod treatment did not affect freezing neither in context A or B (Figure 5c) in mice that did not receive the electric shock during the training day.

## Discussion

The present study strongly suggests that fingolimod, an oral prescription medication for the treatment of MS, is capable of inducing neurogenesis in the adult hippocampus and at the same time improves contextual fear memory. The pro-neurogenic effects appear to be cell autonomous as it was confirmed by *in vitro* experiments in cultures of isolated NS/PCs from postnatal hippocampus that express the S1P receptor type 1, S1P1. Interestingly, a recently published study suggests that quiescent adult NSCs express high levels of GPCRs mRNA, and specifically that of the S1P receptors, compared with active NSCs in the subventricular zone,^26^ implicating S1P in the regulation of stem cells quiescence. Fingolimod, in this case, could force quiescent NSCs to enter cell cycle and proliferate by inducing sustained activation and internalization of S1P1. However, the exact physiological role of S1P has to be extensively researched to decipher that of fingolimod in this particular process.

S1P as well as fingolimod-p’s mitogenic and pro-survival actions in other cell types have been widely discussed in the literature and they seem to be affected by both extra- and intracellular mechanisms.^13, 27^ In agreement with previously published reports,^16, 28^ we found that the induction of GPCR signaling and the concomitant activation of MAPK pathway is responsible for the effects of fingolimod-p on the proliferation of postnatal hippocampal NSCs. Furthermore, by exploring the mechanisms mediating the fingolimod pro-survival effects, we examined the involvement of BDNF, a regulator of NSCs survival both *in vivo* and *in vitro*.^29, 30^ We have found that BDNF is upregulated in postnatal hippocampal NSCs in culture following treatment with fingolimod-p and does indeed mediates the fingolimod-induced protective effects.

Surprisingly, the increase in BDNF production was insensitive to the action of PTX or the MEK1 inhibitor, which lead us to speculate that it may involve a different mechanism other than activation of surface receptors. Indeed, endogenous S1P as well as fingolimod-p binds to histone deacetylase HDAC1 and HDAC2, inhibiting their enzymatic activity and thus enhancing histone acetylation.^31, 32^ Thus, fingolimod appears to be capable of inducing gene expression of various transcription and growth factors including BDNF.

In a subsequent set of experiments, we sought to determine whether the cellular effects of fingolimod on NS/PCs are also ‘translated’ in a significant functional output, in the form of improved contextual discrimination—a hippocampal neurogenesis-dependent memory function.^1, 2, 25, 33^ For this purpose, young mice were subjected to the contextual fear discrimination paradigm that has been used to depict the crucial role of DG and neurogenesis in pattern separation.^25^ Our results show that mice that have been treated with fingolimod displayed an enhanced ability to discriminate between a hostile from a similar environment compared with controls not exposed to the drug. Since defects in pattern recognition and neurogenesis lead to generalized fear, a common feature of anxiety disorders including the post-traumatic stress disorder,^34^ fingolimod could beneficially affect the progress of post-traumatic stress disorder. This has been proposed before by a study, which showed that fingolimod facilitates deletion of established fearful memories; an effect that has been associated to an epigenetic induction of brain plasticity mechanisms.^32^ In addition to the previous study, another recent study has also suggested neurogenesis-related behavioral effects of fingolimod and specifically that confers antidepressant-like activity in stressed animals by inhibiting deacetylase activity.^35^ Although the authors failed to detect any increase in neurogenesis in non-stressed animals, this may still take place because of the differences in experimental design of the two protocols, since this particular group examined neurogenesis 12 days after the end of the last fingolimod injection.

Fingolimod represents an oral therapeutic option for MS, a neuroinflammatory and neurodegenerative disease, where neurogenesis seems to be upregulated possibly as a compensatory mechanism to neuronal loss.^36, 37, 38^ However, it has been shown that newborn cells fail to survive and differentiate to functional neurons at later stages of this disease,^6^ as it has been observed for other neurodegenerative conditions. On the other hand, apart for its neurological clinical findings, MS is also accompanied by disturbances in cognition or mood that are attributed to brain atrophy due to chronic demyelination especially when the hippocampus is being involved.^7, 39^ The deficits observed include difficulties in memory, both in encoding and in retrieval, especially these implicated in information processing,^7, 40^ while brain inflammation itself has been recently shown to have a negative impact on hippocampus ability for pattern separation.^41^ In addition to these, major depression is a common manifestation of the disease.^42^ In this context, fingolimod, might be proved more useful in controlling these negative symptoms, compared with other therapies in MS.

In conclusion, our study is consistent with convergent data suggesting a neurorestorative and regenerative capacity of fingolimod and supports the idea that it may be beneficial in ameliorating the cognitive dysfunction observed in other than MS neurodegenerative conditions, at least in part, through regulation of adult hippocampal neurogenesis.^43, 44, 45^
